# Buruli Ulcer Disease and Its Association with Land Cover in Southwestern Ghana

**DOI:** 10.1371/journal.pntd.0003840

**Published:** 2015-06-19

**Authors:** Jianyong Wu, Petra Tschakert, Erasmus Klutse, David Ferring, Vincent Ricciardi, Heidi Hausermann, Joseph Oppong, Erica A. H. Smithwick

**Affiliations:** 1 Department of Geography, The Pennsylvania State University, University Park, Pennsylvania, United States of America; 2 Ghana Health Service, Accra, Ghana; 3 Department of Geography, Rutgers University, Piscataway, New Jersey, United States of America; 4 Department of Human Ecology, Rutgers University, New Brunswick, New Jersey, United States of America; 5 Department of Geography, The University of North Texas, Denton, Texas, United States of America; University of Tennessee, UNITED STATES

## Abstract

**Background:**

Buruli ulcer (BU), one of 17 neglected tropical diseases, is a debilitating skin and soft tissue infection caused by *Mycobacterium ulcerans*. In tropical Africa, changes in land use and proximity to water have been associated with the disease. This study presents the first analysis of BU at the village level in southwestern Ghana, where prevalence rates are among the highest globally, and explores fine and medium-scale associations with land cover by comparing patterns both within BU clusters and surrounding landscapes.

**Methodology/Principal Findings:**

We obtained 339 hospital-confirmed BU cases in southwestern Ghana between 2007 and 2010. The clusters of BU were identified using spatial scan statistics and the percentages of six land cover classes were calculated based on Landsat and Rapid Eye imagery for each of 154 villages/towns. The association between BU prevalence and each land cover class was calculated using negative binomial regression models. We found that older people had a significantly higher risk for BU after considering population age structure. BU cases were positively associated with the higher percentage of water and grassland surrounding each village, but negatively associated with the percent of urban. The results also showed that BU was clustered in areas with high percentage of mining activity, suggesting that water and mining play an important and potentially interactive role in BU occurrence.

**Conclusions/Significance:**

Our study highlights the importance of multiple land use changes along the Offin River, particularly mining and agriculture, which might be associated with BU disease in southwestern Ghana. Our study is the first to use both medium- and high-resolution imagery to assess these changes. We also show that older populations (≥ 60 y) appear to be at higher risk of BU disease than children, once BU data were weighted by population age structures.

## Author Summary

We studied the relationship between a neglected tropical disease, Buruli Ulcer (BU), and landscape disturbance. We hypothesized that the increased presence of BU was related to landscape disturbance, especially alluvial mining, in endemic areas of the disease. We characterized recent cases of BU in southwestern Ghana and characterized six types of land cover classes at different spatial extents at the village level. We illustrated that mining and water areas were prevalent in BU clustered areas and may potentially play an interactive role in BU occurrence, a finding that should be explored further. No previous study has quantified the relationship between mining and BU at the village level. In addition, we found that people ≥ 60 years old had a higher prevalence than other age groups, when data was weighted by the population age structure. To our knowledge, these findings have not been reported in Ghana before and might provide new insight in BU disease intervention and control. While it is unclear how people come in contact with the bacterial source, our study shows that the importance of where people live (specifically, what land activities are occurring in the area) seems to play a large role in determining disease risk.

## Introduction

Buruli ulcer (BU), a neglected emerging infectious disease, is a skin and soft tissue infection caused by *Mycobacterium ulcerans* (MU). The infection is characterized by painless nodules with necrotizing toxins that produce lesions in the skin, and which may lead to scarring, contractual deformities, amputations, and disabilities if untreated [1–3]. BU has been reported in over 30 countries in West Africa, Southeast Asia, Central and South America and the Western Pacific, as well as Australia [1,4,5]. Though the exact transmission mode of BU disease is still unclear, evidence indicates that the outbreak of BU is associated with climate factors (rainfall and flooding), proximity to slow-flowing or stagnant water, and human-linked environmental disturbance, such as alluvial, pit and sand mining operations, deforestation, and agriculture [2,6–8].

Since different types of land cover classes have different influences on the distribution of human population, the habitat of vectors, and the presence of causative pathogens, examining the association between land cover and BU provides valuable information to prioritize and target specific areas for intervention and control of the disease [8]. The association between land cover and BU has been reported in some countries. In Benin, higher BU disease prevalence rates were found to be associated with rural villages surrounded by forest and at low elevation areas with variable wetness patterns [9]. Similarly, in Australia, the highest BU risk areas were also located at low elevation areas covered by forest [10]. In Côte d'Ivoire, high-risk zones for BU were located in irrigated rice fields, as well as in banana fields and areas in the vicinity of dams used for irrigation and aquaculture [11]. In Cameroon, the Nyong River and associated cultivated wetlands were identified as the major driver of BU incidence [12].

Ghana, located in West Africa, with a population of 24.2 million and a total area of 238,535 km^2^, is one of the most endemic countries of BU disease, second only to Cote d’Ivoire [13]. The first possible BU case in Ghana was reported about 40 years ago [4]. Since then, more BU cases were reported in the country, especially in southern Ghana [14]. In 1993, 1,200 cases were recorded in four regions by a passive surveillance system. A comprehensive national case search in 1999 identified 5,619 BU patients. Based on this data, the national prevalence rate was estimated as 20.7 per 100,000, but up to 150.8 per 100,000 in Amansie West, the most disease-endemic district in Ghana [15]. Since 1999, new BU cases ranged from 326 to 1202 per year, though cases may have been underreported [16].

Based on satellite data, land cover in Ghana has changed dramatically in recent decades [17,18]. Yet, examination of the impacts of land cover and its effect on BU disease has mostly been attempted at district or regional scales [7,19,20], inevitably neglecting local variation in land cover. For example, by analyzing the relationship between land cover and BU disease at the district level using Landsat imagery, Ruckthongsook [20] found that closed-forest areas were positively correlated with BU incidence in southwestern Ghana. In another study, Duker et al. [7] showed that mean BU prevalence was higher in settlements along arsenic-enriched drainages and arsenic-enriched farmlands based on ASTER images. That study examined relationships between the prevalence of BU and several spatial environmental factors in a smaller scale (61 settlements in the Amansie West District), however, it did not examine the association between BU prevalence and land cover.

To date, neither the characteristics of recent BU cases nor the association between BU prevalence and land cover at different scales is well understood in southwestern Ghana. In the present study, we aim to 1) characterize the age and sex patterns of recent BU cases (2007–2010) in southwestern Ghana, 2) illustrate spatial distribution and spatial clusters of BU disease surrounding individual villages, and 3) examine the association between BU prevalence and major types of land cover classes at different spatial extents.

## Methods

### Ethical statement

Approval for this study was obtained from the Institutional Review Board (IRB) at the Pennsylvania State University (PSU), which specified oral informed consent for participation for adults and heads/chiefs of communities as well as implied informed consent for parents on behalf of their children. Assent forms for children <18yrs was also approved by PSU’s IRB. No single individual declined the invitation to participate. In addition, the BU case data were analyzed anonymously and no private information was disclosed in this study.

### Study area

**Fig 1:**
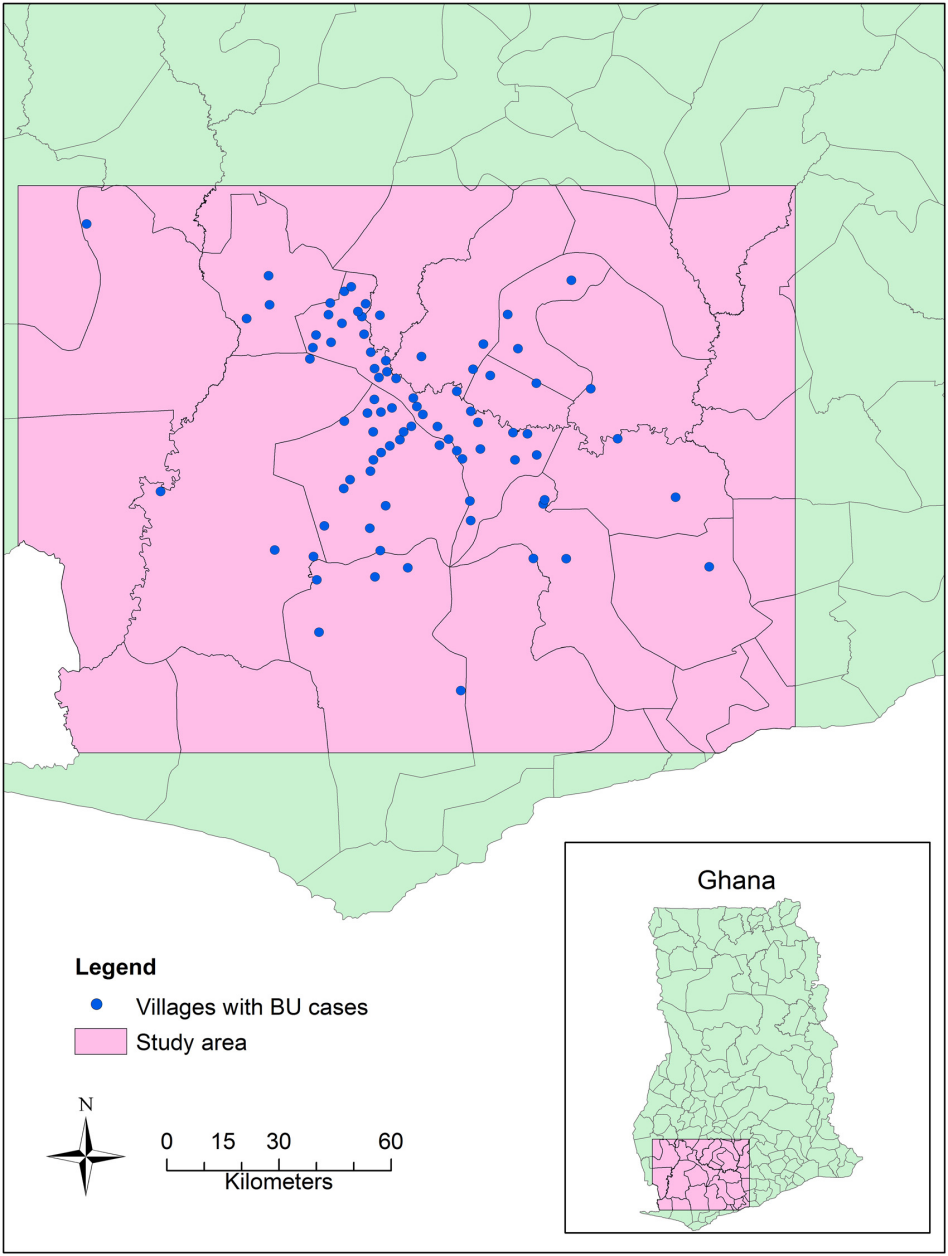
The study extent used to assess the relationship between Buruli Ulcer and land use in southwest Ghana. The study extent was determined by the Landsat images, which covered ~95% of the villages/towns with BU cases in the clinical dataset.

Our study area is in southwestern Ghana, including a large part of Central Region and Western Region, and a small part of Ashanti Region and Eastern Region. For land cover analysis, the whole study area is covered by two Landsat-7 scenes, ranging from 1.00° W to 2.875° W, 5.141° N to 6.515° N (Fig 1). The focus of the study was the Upper Denkyira District, Central Region, where the prevalence of BU is higher. According to the Ghana national census in 2010, the population in Western Region and Central Region is 2,376,021 and 2,201,863, respectively. The sex ratio of male to female is 50:50 and 48:52, respectively.

### Buruli ulcer data collection and analysis

BU case data were collected by district hospitals and clinics mainly located in the Upper Denkyira District, Central Region. This area accounts for the majority of BU cases (>85%) in Central Region and Western Region that was reported by the 2004–2009 Ghana national BU dataset. These hospitals and clinics have good facilities for BU diagnosis and treatment and accept patients mainly from Central Region and Western Region. When patients visit a clinic or hospital, they are diagnosed by experienced doctors to examine whether they have BU based on symptoms. Therefore, these cases were clinically confirmed, rather than laboratory-confirmed. If a case is determined, detailed information about the patients (e.g. age, gender, residence) was recorded. The location of lesions, including the upper limbs, the lower limbs, the head and neck, and other parts were also recorded. According to the date of BU diagnosis, the number of BU cases in each month in the study area was calculated, then its seasonal pattern and annual trend were explored with a seasonal-trend decomposition analysis using the STL function in R package, which decomposed the monthly BU case data into three components: trend, seasonal, and remainder [21].

Based on the residence of these patients, we calculated the number of BU cases in 91 villages [22]. The prevalence of BU disease in these villages was calculated using the observed total numbers of BU cases in a village during 2007–2010 divided by the population of that village. The prevalence of BU disease in the entire study area was calculated using the total number of observed BU cases divided by the total population in the study area (the total population in Central Region and Western Region was used as a proxy). The prevalence of BU in each age and sex group was calculated using the number of BU cases in that age and sex group divided by the total population in that group and expressed as cases per 100,000 people.

### Spatial cluster analysis

Spatial Scan statistics [23] were used to determine whether there were spatial clusters of BU among the villages in the study area. For this analysis, 6 of the 91 villages with BU case information were excluded because they fell slightly outside the study area where land cover was analyzed. The scan statistics approach creates a window (a circle or an ellipse) of point data across space and time, then calculates the observed value and expected value in and outside of the window. The null hypothesis is that the observed value in the window should be equal to that outside of the window. A likelihood ratio test is used to examine whether the cluster is a real cluster or due to a chance [24,25]. The likelihood function is maximized across windows in different locations and sizes, and the window with the maximum likelihood is determined as the most likely cluster. The p value is obtained through a Monte Carlo simulation, which is the rank of the maximum likelihood of the observed value divided by the total number of the maximum likelihood values. For example, if the rank is 10, the simulation number is 999, and the total number of the maximum likelihood values is 1000, then p = 10/1000 = 0.01.

SaTScan v9.1 package was used to detect spatial clusters of BU disease [24,25]. A spatial retrospective analysis with a Poisson probability model was implemented to scan areas with high rates of BU prevalence in 999 Monte Carlo simulations. The total number of BU cases during 2007–2010 in each of the 85 villages was used as the case file, the population in each village in 2010 was used as the population file, and the latitude and longitude of each village was used in the coordinates file.

### Satellite data and image processing

Landsat imagery was used to classify land cover in the study area at a medium spatial resolution (30 m). Two scenes (path 194 and 195, row 56) in 2008 without the coverage of cloud were acquired from the U.S. Geological Survey (USGS) (https://glovis.usgs.gov). The Landsat scenes were radiometrically corrected by transforming digital number (DN) values to reflectance. Bands 1, 2, 3, 4, 5 and 7 of each image were selected and stacked into a new image. Then the images were transformed into brightness, greenness and wetness by the Tasseled cap method [26] and followed by land cover classification. To quantify land cover at a finer spatial scale, we used Rapid Eye imagery (BlackBridge Ltd., Germany) with a resolution of 5 m. Rapid Eye images acquired on January 8, 2012 were first preprocessed by geometric and radiometric correction, then bands 1, 2, 3 were selected for classification.

We based our initial land cover classification scheme on the USGS classification system [27] for both Landsat and Rapid Eye images. This system classifies land use and land cover into nine level 1 classes that could be discerned at both scales. According to the land cover characteristics in southwestern Ghana, we are able to classify urban land, agriculture land, grassland, water and forest. Mining area is commonly classified as a level 2 class under urban land or barren land. Here, we classified mining area separately because it is prevalent and a typical type of land cover class in southwestern Ghana [28]. For example, in the Wassa West District, southwestern Ghana, surface mining expanded from 0.2% of the mining concession areas in 1996 to 49.6% of the concession area in 2002, leading to a substantial loss of forest (58%) and farmland (45%) within mining concessions [28]. In total, we generated six classes and provide detailed information about each class in Table 1. In the Landsat images, the regularly distributed black strips due to the shutdown of the scan-line corrector (SLC-off) were treated as a separate class, termed unclassified.

**Table 1:** Land cover classes used in image classification.

Land cover class	Explanation
Urban	Residential area, road
Mining	Alluvial and hard rock mining
Water	Mining water, reservoirs, lakes, rivers
Grassland	Grassland or similar
Forest	Forest reserves or similar
Agriculture	Cropland, plantations, non-crop trees

We used supervised classification with the maximum likelihood algorithm to create classified maps. The training and testing sites in the classification approach were selected randomly in areas where the land covers were seen to be relatively homogeneous, which was determined using multiple sources of information including a ground truth survey in 2012 as well as Google Earth in 2010, community participatory maps in 2011 and 2012, and high resolution images (e.g. Quickbird images in 2010 with 0.5 m resolution). Land cover classes were initially selected based on participatory mapping activities in each community. Through these mapping activities, community members illustrated their village terrain at the time, including land use and land cover types such as various crop fields, forests, mining areas, and stagnant and flowing water bodies as well as important community infrastructure (e.g. school, wells). During discussions while creating the map, community members also identified areas labeled as “BU risk areas”, indicating polluted or contaminated areas that they considered as possible reservoirs for the MU bacteria and BU transmission (e.g., refuse dumps, areas of stagnant and dirty water in between neighborhoods). In a subsequent mapping activity, community members were encouraged to illustrate changes in land use and land cover over three to four decades, indicating changes in crop lands, areas of deforestation, expanding mining activity, and shifts in areas exposed to flooding. In addition, a ground truth survey included preliminary identification of candidate land covers from an unsupervised classification and field verification of detailed land use/land covers and the associated geographic coordinates. After the supervised classification, isolated classified pixels were removed through the sieving procedure and similar adjacent classified pixels were clumped together through the clumping procedure to smooth these images. The overall accuracy and the Kappa coefficient were used as indices of the classification accuracy, which are derived from the error matrix, a cross-table of the mapped class versus expected class. The image processing was carried out with ENVI 4.8 package (Exelis, Inc., VA, USA).

After classification, we imported the classified images into ArcGIS 10.1 (ESRI, Redlands, CA, USA) for further analysis. First, we converted raster layers into shapefiles. Where Rapid Eye and Landsat images overlapped, land cover classes from the Rapid Eye image were used; if Rapid Eye image was not present, land cover classes from the Landsat image were used. We created a buffer around each village with the radius as 1 km, 2.5 km, 5 km, 10 km, 20 km and 30 km and 40 km, respectively. We calculated the area of each land cover class in each buffer by intersecting each buffer with the classified image [22]. The percentage of each land cover was calculated by dividing its area by the total area of the buffer (unclassified land cover areas were included in the total area). In the same way, the percentage of each land cover class for 154 villages/towns, including 85 villages where BU cases were reported and 69 villages randomly selected as the control, where BU cases were not reported. To examine the effect of unclassified areas on the quantification of the land cover classes, mainly caused by the scan line off (SLC-off) problem, we used a modified nearest neighbor approach [29]. Specifically, we replaced the value of unclassified pixels with the average value of the left neighbors and right neighbors, respectively, and then averaged the results to obtain a new land cover class, essentially filling the gaps caused by the SLC-off problem.

### Statistical analysis

To compare the number of BU cases by different gender and age groups, the Friedman test, a non-parametric equivalent of two-way ANOVA, was used considering the BU data could not be assumed to follow a normal distribution. In this analysis, the dependent variable was the BU cases, the two influential factors were gender and age group. Gender has two levels: male and female; and age group has three levels: 0–19 yrs, 20–59 yrs, and > 60 yrs. To examine the difference of the percentages of land cover types in the BU clustered area and the whole study area, Wilcoxon signed-rank test, a nonparametric equivalent of Paired t-test was used because the percentages of land cover types around villages were not assumed to follow a normal distribution. These tests were conducted with SAS 9.3 (SAS Institute, Inc, Cary, NC, USA).

To examine the association between BU prevalence and each land cover class, a set of regression models, including Poisson and negative binomial regression models, were developed [8]. In these models, we used the number of BU cases in each village as the dependent variable, the natural logarithm of the population of each village as the offset term, and the percentage of each land cover class as independent variables. Since we had six categories of land cover, we had six independent variables in the model.

Initially, we assumed the count data (BU cases, expressed as Y) followed a Poisson distribution, of which the mean, E(Y), and the variance, Var (Y), are assumed equal (E(Y) = Var (Y) = μ). Goodness of fit test using the deviance showed that Poisson distribution was not a good fit (deviance/ degree of freedom >3.00). Therefore, we selected a negative binomial distribution, which allows the variance is higher than the mean (E(Y) = μ, Var(Y) = μ +kμ^2^, k is an overdispersion coefficient). The general equation of the negative binomial regression model is written as below:

$$\text{log}\left(\mu_{i}\right)=\text{log}\left(N_{i}\right)+\;\beta_{0}+\beta_{1}x_{1i}+\beta_{2}x_{2i}+\ldots +\;\beta_{n}x_{ni}$$ (1)

Where μ is the expected number of BU, i is the index of an individual village, N is the number of population of a village, x_1_, x_2_, … x_n_ are the covariates of the model, denoting the percentages of land cover classes, respectively. The summary statistics of these covariates were listed in S1 Table. Considering the interactive effects between water and mining as well as water and agriculture, we put two interaction terms into the model, namely, the percentage of water area multiplied by the percentage of mining area, and the percentage of water area multiplied by the percentage of agriculture area. Before running the model, we examined multicollinearity with correlation matrix generated by Pearson correlation analysis and variance inflation factors (VIF) calculated by SAS reg procedure. If mulitcollinearity was found among covariates (e.g. r >0.6 or VIF>5), only one of these highly correlated covariates was put into the model. Akaike’s Information (AIC) as the criterion was used to select the best fitted models. A smaller AIC value indicates a better fitted model. If the regression coefficient (β) for a land cover class was significantly larger than zero (β>0, p<0.05), we assumed the prevalence of BU had a positive association with that land cover class. If β was significantly less than zero (β<0, p<0.05), a negative association was assumed. To evaluate model performance, we ran residual diagnostics of the top-rank model. We calculated the predicted value, the raw residual, the deviance residual, the standardized Pearson residuals, standardized deviance residual and likelihood residual for each observation and mapped the standardized Pearson residuals and the standardized deviance residual to illustrate the model performance. The raw residual, deviance residual, the standardized Pearson residuals and the standardized deviance residual were also assessed for spatial dependence with global Morans’ I, an index of spatial autocorrelation. The model fitting and residual diagnostics were carried out using the SAS genmod procedure with SAS 9.3 (SAS Institute, Inc., Cary, NC, USA) and spatial autocorrelation was measured with ArcGIS 10.1 (ERSI, Redlands, CA, USA)

To consider spatial dependence in residuals, a spatial lag model was developed to examine the association between BU cases and the percentages of land cover classes. For the spatial lag model, the dependent variable at village i is assumed to be affected by the neighbors of village i. A general equation for the spatial lag model was shown below [30]:

$$\text{log}\left(y_{i}\right)=\rho \text{W}*\text{log}\left(y_{i}\right)+\beta_{0}+\beta_{1}x_{1i}+\beta_{2}x_{2i}+\ldots +\;\beta_{n}x_{ni}$$ (2)

Where, the dependent variable (log (y_i_)) is the natural logarithm transformed BU prevalence in the unit of case per 100,000 people, i is the index of the villages, W is the spatial weight matrix, ρ is the spatial autoregressive coefficient, β_1_… β_n_ are the regression coefficients of covariates, and x_1_…x_n_ are the percentages of land cover classes. The covariates in the spatial lag model are same as that in the top-ranked negative binomial models. The spatial weight matrix W was created based on the Euclidean distance among villages. After fitting the model, the normality and spatial autocorrelation of model residuals were assessed using Jarque-Bera test and Morans’ I, respectively. The model fitting and residual diagnostics were carried out with GeoDa 1.6.6 package [31].

## Results

### Characterization of BU cases

**Fig 2:**
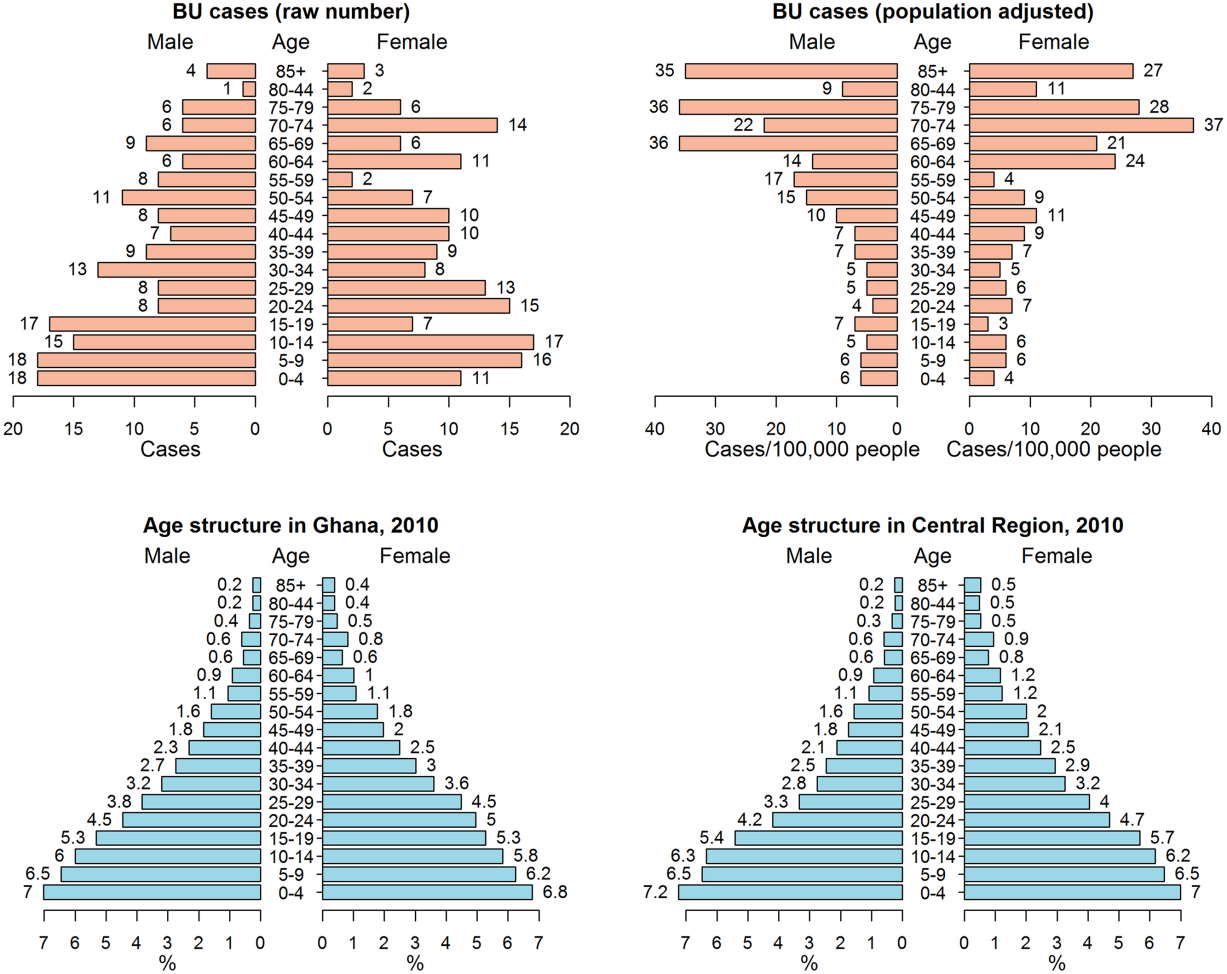
Age and sex of hospital-collected BU cases and population age structure in southwestern Ghana. Upper left: The distribution of BU diseases by age and sex; upper right: The adjusted distribution of BU prevalence by age and sex. The age structure in Ghana in 2010 was used to adjust BU cases in each age and sex group; bottom left: the population age structure in Ghana in 2010; bottom right: the population age structure in Central region, Ghana, in 2010.

Of the 339 BU cases reported in the study area during 2007–2010, 172 cases were male and 167 cases were female. BU cases were found in all age groups (Fig 2). The cases among children < 5 years account for 8.6%, age 5–14 account for 19.8%, age 15–34 account for 26.3%, while BU cases among the older population (age ≥ 60 y) account for 22.1%. The Friedman test on this raw data showed that the number of BU cases among older groups was significantly lower than that among the other groups under 60 (p < 0.01) and there was no difference between the numbers of male cases and female cases (p > 0.05). However, when the number of BU cases was adjusted by population age structure (the number of the observed BU cases in a specific age or gender group divided by the population in that group) (Fig 2), the prevalence of BU disease was significantly higher for the older population (age ≥ 60) when compared with population under 60 (p < 0.01).

**Fig 3:**
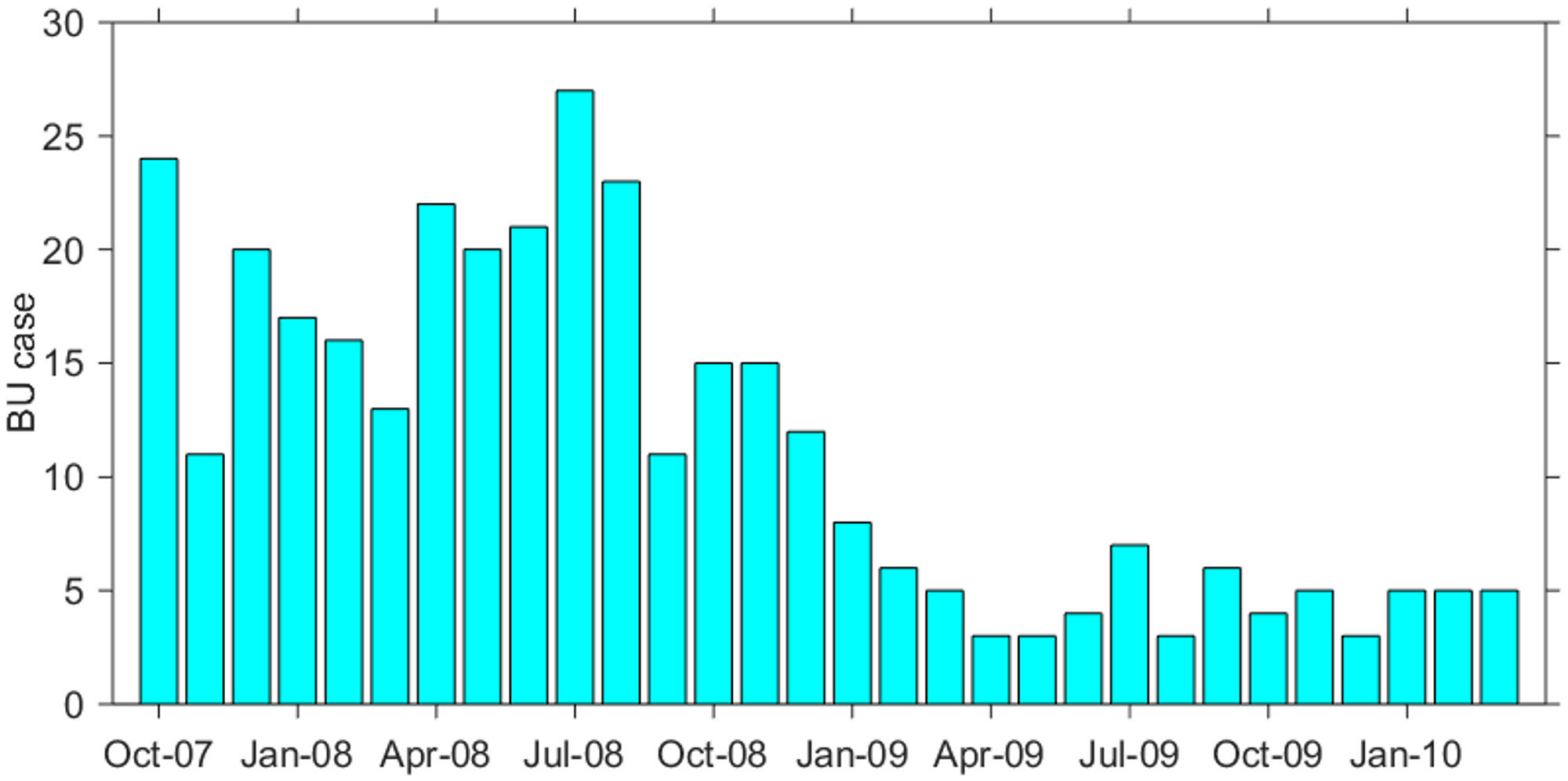
Monthly variation of BU cases during 2007–2010. BU cases were collected from October 2007 to March 2010. There were 11.3 cases in each month on average and the highest number of BU (27 cases) was observed in July 2008.

BU cases were reported in each month, making it possible to detect temporal trends (Fig 3). The number of BU cases was slightly higher in July. It also varied across years and was nearly 3.7 times higher in 2008 than that in 2009. The season-trend decomposition analysis also showed that the number of BU cases had a seasonal pattern, which was higher in summer season (June-August, peak at July), and lower in other seasons, and there was a decreasing trend by year (S1 Fig). In terms of the location of the disease on the human body, 74% of lesions were found in lower limbs and 21.5% were found in upper limbs.

### Spatial distribution and cluster of BU disease

Of the 85 villages in which BU cases were used for spatial analysis, most were located in Central Region and Western Region. The Upper Denkyira District in Central Region and Wasa Amenfi East District in the Western Region have the most BU cases, which accounted for 62.2% and 15.3% of the total cases, respectively. Some individual villages/towns such as Dunkwa, Dominase, Ayanfuri, Jameso Nikwanta, and Maudaso had a high number of BU cases (>10 cases).

**Fig 4:**
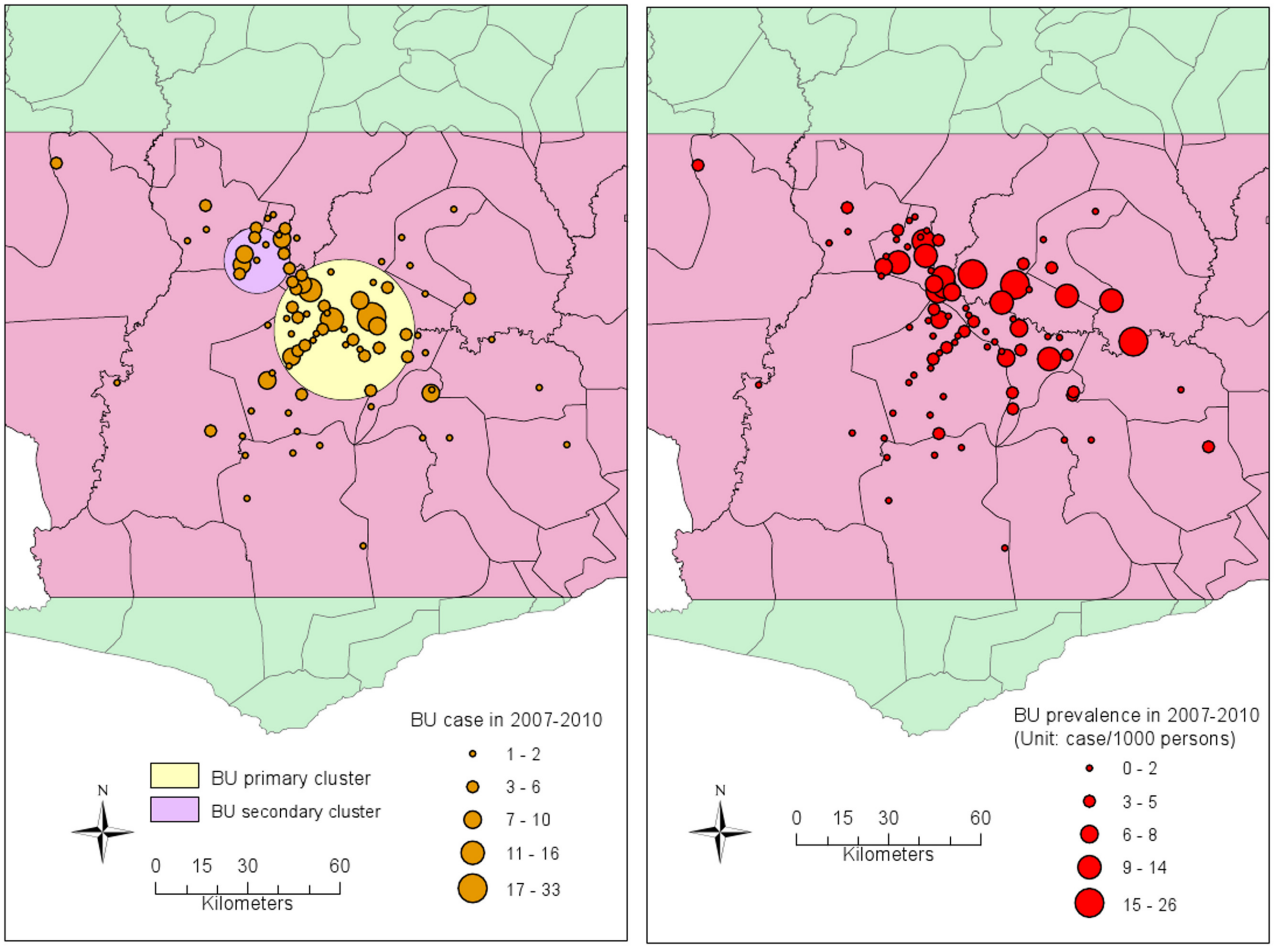
Spatial distribution and spatial cluster of BU cases and prevalence. The map to the left shows the spatial distribution of BU cases in each village/town. The large yellow circle is the primary cluster of BU disease, which is located at 5.930°N and 1.861°W with a radius of 22.94 km. It includes 33 villages and 174 BU cases. The map to the right is the spatial distribution of BU prevalence in each village/town. The prevalence is calculated using the number of BU cases divided by the population in each village.

The spatial scan analysis identified two significant BU clusters (the number of BU cases > 10; p < 0.01) in southwestern Ghana, one primary cluster and one secondary cluster. The primary cluster was located at 5.930°N and 1.861°W with a radius of 22.94 km. It included 33 villages and 174 BU cases (Fig 4). The secondary cluster was located at 6.135° N and 2.118° W with a radius of 10.75 km, which includes 9 villages and 45 BU cases (Fig 4).

### Association of BU with land cover

**Fig 5:**
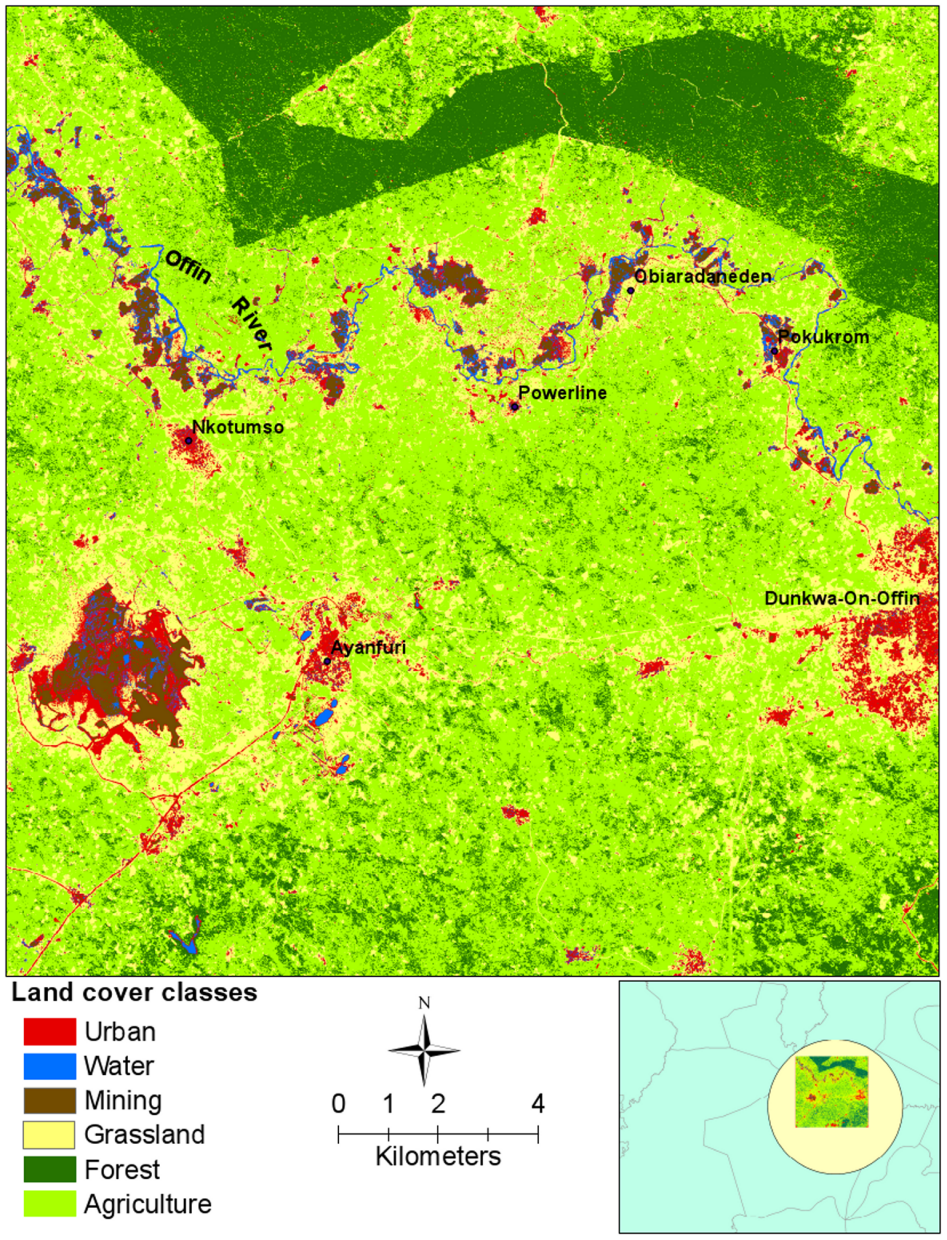
Land cover map of the primary BU cluster based on the classification of the Rapid Eye image. Bottom inset shows extent of satellite imagery within the cluster. The area has a few villages/towns with a high number of BU cases, e.g. Ayanfuri and Pokukrom.

**Fig 6:**
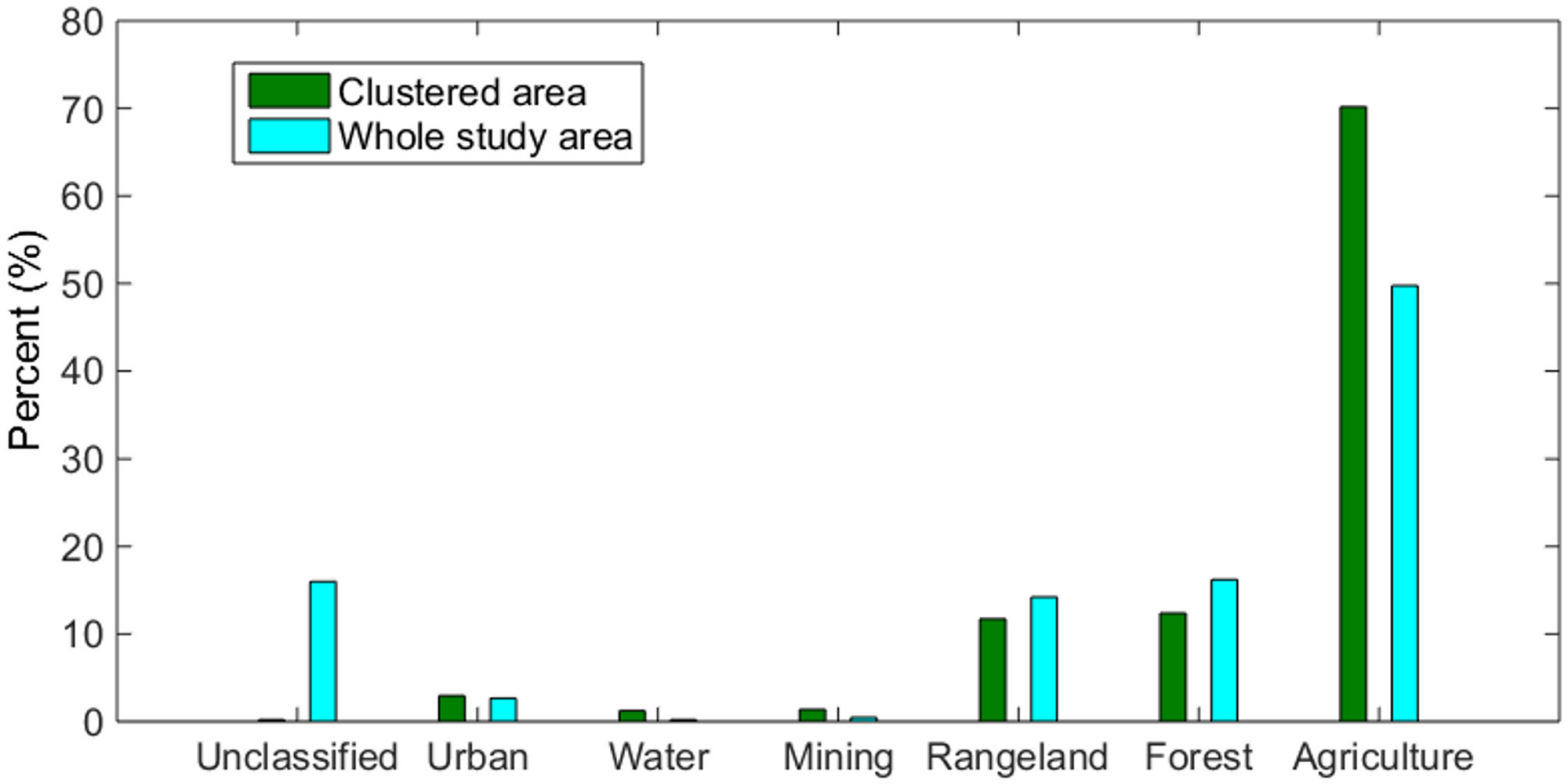
Comparison of land cover in the BU clustered area (574 km^2^) with the whole study area (31,580 km^2^). Here, the clustered area denotes the area covered by the Rapid Eye image in the primary cluster of BU disease as shown in Fig 5; the whole area is the area covered by the Landsat image as shown in Fig 1.

The overall accuracy of the land cover classification for the high resolution (Rapid Eye) and medium resolution (Landsat) was 93.4% and 82.2%, respectively. Based on the Rapid Eye image, the major type of land cover class is agriculture (70.2%), followed by forest (12.4%) and grassland (11.7%). Water and mining areas are relatively small, only accounting for 1.0–1.5% (Figs 5 and 6). The classified Rapid Eye image showed that many small scale mining patches were distributed along the river edge or near adjacent water areas (Fig 5).

The types of land cover classes in the entire study area covered by the Landsat images (as shown in Fig 1) are similar as those in the area covered by the Rapid Eye image in the BU clustered area. However, the percentages of water, mining and agriculture areas in the entire study area were smaller than these in the BU clustered area, especially for water and mining areas (Fig 6). Wilcoxon signed-rank test showed that land cover components were not significantly different between the two spatial extents (the entire study area vs. the BU clustered area, p>0.05). In the entire study area, there is nearly 16% of land cover in the unclassified category, largely due to the dysfunction of the satellite detector.

The results of the final negative binomial regression model based on AIC values are presented in Table 2, showing that associations between BU prevalence and the percentages of land cover classes at the village level varied as a function of buffer distances (around a village/town). Overall, the percentage of urban area had a significantly negative association (p<0.01) with BU prevalence in all distances from 1 km to 40 km with the mean regression coefficient ranging from-2.109 to -0.035, indicating that more urbanized villages might have a low risk of BU prevalence. The percentage of water area had a positive association with BU prevalence in the distances from 1 km to 20 km and associations were significant except that at 1 km, of which p value was slightly above 0.05. The mean regression coefficient for the water variable ranged from 0.224 to 2.950 and was larger than these of other covariates, which suggested that the increase in water area in a buffer in 2.5–20 km might lead to a large increase in BU prevalence. However, when the distance was 30 km and 40 km, the positive association between water and BU was not held. Instead, the percentage of mining area showed a strong positive association with BU (p<0.01, β = 3.266–4.195). The percentages of grassland and agriculture also had positive associations with BU prevalence. The association for grassland area was significant at all distances, and the association for agriculture was significant at 30 km to 40 km. The increases in grassland and agriculture areas might slightly increase BU prevalence in some spatial extents, as indicated by the regression coefficients of both covariates, which ranged from 0.014 to 0.136 and 0.091 to 0.111, respectively. The association between the percentage of forest and BU prevalence varied as the change of the buffer distances, which was positive at the distances of 1, 2.5 and 5km, and was negative at the distances of 20, 30 and 40km, respectively. The association was significant only at 2.5 km and 5 km. Two interaction terms, water with mining and water with agriculture, were not selected in the final model based on AIC, likely because the term was strongly correlated with individual land covers (S2 Table). The evaluation of model performance for the final model with the smallest AIC value (distance = 30 km) showed that the model fitted the data very well, indicated by very small standardized Pearson residuals and standardized deviance residuals (S2 Fig). The examination of Moran’s I showed that both standardized residuals had no significant spatial autocorrelation, while the raw residuals were spatially correlated.

**Table 2:** The association of BU prevalence and land cover classes at different spatial extents using negative binomial regression models. The dependent variable is the natural logarithm-transformed number of BU cases in each village with the natural logarithm-transformed population in each village as an offset. The covariates are the percentages of land cover classes in a buffer in different distances. For each buffer distance, the results of the model with a smaller AIC value were presented.

Buffer radius	Covariates	β	95% CI		p	AIC
			Lower	Upper		
1 km	Urban	-0.035	-0.055	-0.015	0.001	590.60
	Water	0.224	-0.008	0.457	0.059	
	Grassland	0.014	0.003	0.024	0.009	
	Forest	0.025	-0.006	0.055	0.110	
2.5 km	Urban	-0.074	-0.109	-0.039	<0.001	588.15
	Water	0.445	0.082	0.808	0.016	
	Grassland	0.014	0.004	0.024	0.007	
	Forest	0.025	0.001	0.049	0.043	
5 km	Urban	-0.176	-0.254	-0.097	<0.001	579.85
	Water	1.013	0.440	1.585	0.001	
	Grassland	0.016	0.006	0.026	0.003	
	Forest	0.027	0.006	0.049	0.014	
10 km	Urban	-0.437	-0.596	-0.278	<0.001	571.24
	Water	1.778	0.886	2.671	<0.001	
	Grassland	0.019	0.007	0.030	0.002	
20 km	Urban	-1.378	-1.784	-0.971	<0.001	561.20
	Water	2.950	1.874	4.025	<0.001	
	Grassland	0.024	0.010	0.038	0.001	
	Forest	-0.021	-0.068	0.027	0.397	
30 km	Urban	-2.109	-2.787	-1.432	<0.001	527.39
	Mining	4.195	2.545	5.845	<0.001	
	Grassland	0.119	0.081	0.157	<0.001	
	Forest	-0.074	-0.162	0.015	0.103	
	Agriculture	0.091	0.058	0.124	<0.001	
40 km	Urban	-1.686	-2.534	-0.838	<0.001	537.11
	Mining	3.266	1.193	5.338	0.002	
	Grassland	0.136	0.097	0.174	<0.001	
	Forest	-0.095	-0.205	0.016	0.093	
	Agriculture	0.111	0.077	0.145	<0.001	

The dependent variable and covariates in two top-ranked negative binomial regression models based on the AIC values were re-examined by spatial lag models. In the 30 km buffer radius, the percentages of mining, grassland and agriculture areas had positive associations with BU prevalence, and urban and forest areas had a negative association. In the 40 km buffer radius, the percentages of mining, grassland and agriculture areas also had significantly positive associations with BU prevalence, and the percentage of urban area was negatively associated with BU prevalence, however, the association between forest and BU prevalence was not significant. (Table 3).

**Table 3:** The association of BU prevalence and land cover classes in different spatial extents using spatial lag regression models. The two models with smaller AIC values from the negative binomial regression analysis were selected for spatial lag regression analysis. The dependent variable is the natural logarithm -transformed number of BU cases in each village. The covariates are the percentages of land cover classes in a buffer in different distances.

Buffer radius	Covariates	β	95% CI		p	AIC
			Lower	Upper		
30 km	Urban	-1.719	-2.300	-1.138	<0.001	648.96
	Mining	4.509	2.667	6.350	<0.001	
	Grassland	0.070	0.023	0.116	0.003	
	Forest	-0.082	-0.154	-0.011	0.025	
	Agriculture	0.058	0.020	0.097	0.003	
40 km	Urban	-0.878	-1.476	-0.281	0.004	668.67
	Mining	2.911	0.633	5.188	0.012	
	Grassland	0.064	0.016	0.111	0.008	
	Forest	-0.059	-0.158	0.040	0.242	
	Agriculture	0.058	0.017	0.100	0.006	

## Discussion

In this study, we characterized recent cases of BU in southwestern Ghana, identified BU clusters, quantified land cover at two different spatial resolutions (Rapid Eye and Landsat), and evaluated the association between BU prevalence and six types of land cover classes at different spatial extents at the village level. We found that the older population (age ≥ 60) has a higher prevalence than other age groups, indicating higher vulnerability to BU disease. We illustrated that mining and water areas were prevalent in the BU clustered area with high resolution satellite imagery. Moreover, we revealed that urban, water, grassland and mining areas were strongly associated with BU prevalence in the area. While the importance of mining has been independently proposed as key factors, no previous study has quantified their effects at the village level. To our knowledge, these findings have not been reported in Ghana before and might provide new insight in BU disease intervention and control.

It is known that BU disease affects people of all age groups. However, the age group most associated with BU is still debated. Several studies on BU in African countries showed that children less than 15 years of age had a higher risk for BU [6,32,33], while in southeastern Australia, people >60 years of age were associated with a higher rate of BU [34]. There are also some studies showing that both young children and old adults could have a higher risk for BU [35–37]. Our results showed that young children (< 20 y) had a higher number of BU cases in contrast to older people. However, after adjusting disease rates by population age structure, we revealed that older people (≥ 60 y) had a higher risk of contracting BU. Our results suggested that a higher number of BU among children in Africa reported in previous studies did not support that young children were a high-risk group for BU disease, because younger children account for a higher percentage of total population than other age groups while older people account for a lower percentage (Fig 2). Our results from Ghana thus indicate that older males had the highest risk for BU disease, which is consistent with the conclusion by Debackert et al. [36] after adjusting for age distribution.

Using spatial scan statistics, we identified two BU disease clusters, one primary cluster with 174 BU cases and one secondary cluster with 45 cases. The prevalence rates in both clusters are greater than average prevalence rates. The primary cluster covered the main part of upper Denkyira East District, east part of Wasa Amenfi West District, and south part of Obuasi Municipal, Amansie Central and Amansie West District, where a higher prevalence of BU has been observed [15]. The identification of disease clusters helps us target the specific area with higher prevalence and form hypotheses on the relationships between land cover and BU disease. With the high resolution satellite images, many alluvial gold mining patches distributed along the Offin River were evident throughout the BU clustered area. After classifying satellite images, we also found that the percentages of water and mining areas in the BU clustered area were much higher than those in the whole study area. These particular characteristics of land cover in the BU clustered area gave a strong indication that water and mining areas are related to BU disease.

Our results affirm the association between and human modification of aquatic ecosystems [1–3,12,38]. Specifically, the regression coefficients for water as a land cover type were statistically significant at the scales from 1 km to 20 km buffers, while at the 30 km and 40 km buffer scales, the association of BU with mining was significant. In addition, there was a strong correlation between the percentages of water and mining areas, and the interaction term of BU and mining was significant in several competitive models (S3 Table), though not in the final model. These results indicate that water and mining play an important and potentially interactive role in BU occurrence, a finding that should be explored further. Importantly, the high resolution satellite images show evidence of alluvial gold mining along the Offin River. Alluvial mining may promote the formation of stagnant waters that might provide favorable environments for the disease [39]. Other studies have provided evidence for strong associations of BU with main rivers systems and disturbed water bodies [1,2,6,7,12]. For example, Landier et al.[12] showed recently that the Nyong River was the major driver of BU incidence in Cameroon, which they attributed to wetland presence, cultivation, and forest clearing. In the case of our study, mining was the primary cause of human modification of the alluvial environment. Ours is the first study to explore the fine-scale relationship between alluvial mining and BU statistically and the results suggest that water and human activity may both contribute to increased BU disease risk.

Agriculture and grassland were positively associated with BU prevalence, indicating that mining may not be the only factor contributing to the disease patterns. Marston et al. [25] showed that participating in farming activities near rivers was a risk factor for BU infection in the Daloa region of Côte d'Ivoire [40]. In Benin, farmers accounted for a larger percentage of case-patients compared to controls and female farmers were associated with increased risk for BU [37]. Generally, as with mining, agriculture might increase nutrient and lower dissolved oxygen in water, the environmental condition facilitating the growth of *M*. *ulcerans* [1]. The grassland land cover category likely includes mixed agricultural systems that could not be binned into agricultural or other land use classes. Our preliminary water quality tests in the study area [41] did not find significant correlations between BU and nutrient or oxygen conditions in water, but did show significant increase in the concentration of heavy metals in water bodies associated with mining activities. Further studies are needed to explore the specific environmental conditions associated with specific types of land use and land cover changes.

Negative correlations between forest area and BU prevalence were expected because other studies have showed that deforestation was associated with BU [2,39]. In general, deforestation can reduce riparian cover and increase water temperature, thus facilitating pathogen growth [42]. It is also thought that through deforestation, *M*. *ulcerans* might be washed into the aquatic environment, which could facilitate its growth and proliferation [39]. However, it was also reported that forest might have a positive association with BU prevalence, e.g., the relationship between BU prevalence and forest in Benin [8]. In our study, the negative association between the percentage of forest area and BU prevalence was not significant and there were positive associations, suggesting the relationship between forest and BU might be complicated.

Finally, urban land cover was negatively correlated with BU prevalence, which is consistent with some studies in Benin [8,9]. As explained in these studies, villages with a larger percent of urban land cover may have better resources to prevent BU disease and better employment opportunities outside of agriculture and/or mining to lower contact rates with high risk habitats [8,9].

Our study has a few limitations. First, similar to many other studies [2,6,8,12,37,43,44], it is likely that the number of BU cases analyzed here is underestimated because if BU patients did not visit hospitals or clinics, we would not have those records. Second, due to the coarse resolution (30 m) of Landsat images, we used a conserved land use classification across both sets of imagery. We do not exclude the possibility that some subclasses of land cover, e.g. cocoa farms, rubber and palm plantations, which are common in the area, might have significant associations with BU prevalence. Similarly, we cannot capture small water patches with the Landsat images, which may underestimate the percentage of water area in the whole study area. For the Landsat image, nearly 16% of the land cover was not classified, mainly caused by the scan line issue. However, the results from the simple gap-filling method showed the land cover assignments before and after the assignment of unclassified area were very similar, suggesting a limited influence on final results (S3 Fig). However, another potential issue is that we used satellite imagery at two different spatial resolutions to quantify land cover surrounding villages. For a few villages, quantification of land cover classes in a buffer might contain satellite data from either the high resolution image or medium resolution image, or both. However, we expect this effect to be minor because the area of the land cover classified with Rapid Eye image only accounted for 1.8% of the total study area. Therefore, in 98.2% of the study area, we used Landsat. Use of high spatial resolution images across the whole study might be better to illustrate the relationship between BU disease and land cover but these images were not available for the entire study area.

The significant association between BU prevalence and land cover does not suggest that land cover change and BU disease have a cause-effect relationship. The land cover change may pose an important but indirect role on BU disease through its impacts on human activities, vector habitat and pathogen distribution. Future studies should investigate how land cover change affects the occurrence of BU disease through landscape pattern analysis and participatory community-based surveys, and examine other risk factors in the area of inquiry, such as socioeconomic status, and environmental and climatic factors. Based on our results, we also suggest that the environmental characteristics of alternative land uses (e.g., mining, agriculture, and their interaction) on water quality in alluvial environments in BU endemic areas is important for understanding spatial patterns of the disease.

## Supporting Information

S1 Table Summary statistics of land cover classes in different buffer distances. (DOCX)

S2 Table The result of Pearson correlation between the percentages of each individual land cover class with two interaction terms. (DOCX)

S3 Table The association of BU prevalence and the interaction between water and mining at different spatial extents using negative binomial regression models. (DOCX)

S1 Fig Seasonal-trend decomposition analysis of monthly BU cases. (DOCX)

S2 Fig The standardized Pearson residual and the standardized deviance residual from negative binomial regression model with a buffer radius of 30 km. (DOCX)

S3 Fig Comparison of land cover components before (plot A) and after (plot B) the assignment of unclassified pixels. (DOCX)
