# Supplementary material for: Buruli Ulcer Disease and Its Association with Land Cover in Southwestern Ghana
Source: PLoS Negl Trop Dis. 2015 Jun 19;9(6):e0003840. doi: 10.1371/journal.pntd.0003840 (PMC4474842; doi:10.1371/journal.pntd.0003840)
Supplement: S1 Table — (DOCX) [file pntd.0003840.s001.docx]

**Table S1.** Summary statistics of land cover classes in different buffer distances.

| **Buffer radius** | **Variable** | **Mean** | **Standard Deviation** | **Minimum** | **Maximum** | **Coefficient of Variation** |
| --- | --- | --- | --- | --- | --- | --- |
| **1 km** | Urban | 11.32 | 15.18 | 0.00 | 94.83 | 1.34 |
|  | Water | 0.22 | 1.12 | 0.00 | 10.69 | 5.03 |
|  | Mining | 0.67 | 3.14 | 0.00 | 34.48 | 4.67 |
|  | Grassland | 23.07 | 27.69 | 0.00 | 94.64 | 1.20 |
|  | Forest | 1.98 | 8.06 | 0.00 | 79.23 | 4.07 |
|  | Agriculture | 49.66 | 32.38 | 0.00 | 99.99 | 0.65 |
| **2.5 km** | Urban | 4.14 | 7.24 | 0.00 | 66.45 | 1.75 |
|  | Water | 0.17 | 0.80 | 0.00 | 7.43 | 4.62 |
|  | Mining | 0.49 | 1.55 | 0.00 | 12.53 | 3.16 |
|  | Grassland | 25.10 | 28.32 | 0.00 | 94.63 | 1.13 |
|  | Forest | 4.45 | 10.35 | 0.00 | 79.32 | 2.32 |
|  | Agriculture | 52.52 | 32.52 | 0.00 | 99.85 | 0.62 |
| **5 km** | Urban | 2.43 | 3.48 | 0.01 | 28.14 | 1.43 |
|  | Water | 0.15 | 0.49 | 0.00 | 3.03 | 3.37 |
|  | Mining | 0.48 | 1.68 | 0.00 | 18.24 | 3.46 |
|  | Grassland | 25.22 | 27.47 | 0.00 | 85.28 | 1.09 |
|  | Forest | 8.32 | 11.92 | 0.00 | 71.69 | 1.43 |
|  | Agriculture | 50.16 | 30.53 | 0.00 | 99.88 | 0.61 |
| **10 km** | Urban | 1.99 | 2.26 | 0.18 | 12.59 | 1.13 |
|  | Water | 0.12 | 0.31 | 0.00 | 1.66 | 2.61 |
|  | Mining | 0.37 | 0.74 | 0.00 | 6.06 | 1.98 |
|  | Grassland | 24.08 | 24.71 | 0.00 | 73.22 | 1.03 |
|  | Forest | 12.38 | 10.68 | 0.01 | 62.13 | 0.86 |
|  | Agriculture | 47.17 | 27.09 | 0.20 | 97.79 | 0.57 |
| **20 km** | Urban | 1.66 | 1.33 | 0.18 | 6.65 | 0.80 |
|  | Water | 0.13 | 0.29 | 0.00 | 2.39 | 2.20 |
|  | Mining | 0.31 | 0.36 | 0.00 | 1.76 | 1.16 |
|  | Grassland | 22.15 | 21.48 | 0.00 | 68.79 | 0.97 |
|  | Forest | 15.09 | 7.45 | 0.03 | 48.82 | 0.49 |
|  | Agriculture | 43.96 | 22.91 | 1.78 | 88.95 | 0.52 |
| **30 km** | Urban | 1.55 | 1.00 | 0.25 | 5.02 | 0.65 |
|  | Water | 0.12 | 0.21 | 0.00 | 1.06 | 1.73 |
|  | Mining | 0.29 | 0.29 | 0.01 | 1.24 | 1.01 |
|  | Grassland | 21.26 | 19.02 | 0.00 | 58.67 | 0.89 |
|  | Forest | 15.35 | 5.68 | 0.96 | 31.28 | 0.37 |
|  | Agriculture | 42.27 | 20.00 | 6.22 | 80.62 | 0.47 |
| **40 km** | Urban | 1.58 | 0.76 | 0.31 | 4.37 | 0.48 |
|  | Water | 0.09 | 0.13 | 0.00 | 0.60 | 1.45 |
|  | Mining | 0.31 | 0.23 | 0.01 | 0.74 | 0.73 |
|  | Grassland | 20.37 | 16.40 | 0.02 | 52.30 | 0.81 |
|  | Forest | 14.74 | 4.82 | 1.86 | 27.54 | 0.33 |
|  | Agriculture | 41.50 | 17.20 | 11.02 | 72.82 | 0.41 |
