# Supplementary material for: Buruli Ulcer Disease and Its Association with Land Cover in Southwestern Ghana
Source: PLoS Negl Trop Dis. 2015 Jun 19;9(6):e0003840. doi: 10.1371/journal.pntd.0003840 (PMC4474842; doi:10.1371/journal.pntd.0003840)
Supplement: S2 Table — (DOCX) [file pntd.0003840.s002.docx]

**Table S2.** The result of Pearson correlation between the percentages of each individual land cover class with two interaction terms.

| Buffer radius |  |  | Urban | Water | Mining | grassland | Forest | Agriculture |
| --- | --- | --- | --- | --- | --- | --- | --- | --- |
| 1km | Water × mining | r | -0.015 | 0.942 | 0.187 | 0.018 | -0.031 | 0.002 |
|  |  | p | 0.850 | <0.001 | 0.020 | 0.826 | 0.701 | 0.984 |
|  | Water × agriculture | r | -0.051 | 0.962 | 0.175 | 0.015 | -0.035 | 0.045 |
|  |  | p | 0.531 | <0.001 | 0.030 | 0.853 | 0.663 | 0.583 |
|  |  |  |  |  |  |  |  |  |
| 2.5 km | Water × mining | r | -0.013 | 0.940 | 0.333 | 0.011 | -0.057 | 0.014 |
|  |  | p | 0.872 | <0.001 | <0.001 | 0.888 | 0.486 | 0.868 |
|  | Water × agriculture | r | -0.006 | 0.955 | 0.324 | -0.017 | -0.062 | 0.067 |
|  |  | p | 0.942 | <0.001 | <0.001 | 0.836 | 0.446 | 0.408 |
|  |  |  |  |  |  |  |  |  |
| 5 km | Water × mining | r | 0.053 | 0.903 | 0.390 | 0.002 | -0.111 | 0.071 |
|  |  | p | 0.513 | <0.001 | <0.001 | 0.979 | 0.170 | 0.380 |
|  | Water × agriculture | r | 0.057 | 0.942 | 0.371 | -0.019 | -0.098 | 0.101 |
|  |  | p | 0.483 | <0.001 | <0.001 | 0.816 | 0.227 | 0.211 |
|  |  |  |  |  |  |  |  |  |
| 10 km | Water × mining | r | -0.018 | 0.922 | 0.420 | 0.020 | -0.045 | 0.044 |
|  |  | p | 0.826 | <0.001 | <0.001 | 0.804 | 0.581 | 0.589 |
|  | Water × agriculture | r | 0.004 | 0.905 | 0.409 | -0.007 | -0.070 | 0.103 |
|  |  | p | 0.959 | <0.001 | <0.001 | 0.931 | 0.385 | 0.202 |
|  |  |  |  |  |  |  |  |  |
| 20 km | Water × mining | r | 0.342 | 0.977 | 0.450 | 0.252 | -0.169 | -0.178 |
|  |  | p | <0.001 | <0.001 | <0.001 | 0.002 | 0.036 | 0.027 |
|  | Water × agriculture | r | 0.152 | 0.875 | 0.402 | 0.111 | -0.083 | -0.006 |
|  |  | p | 0.060 | <0.001 | <0.001 | 0.169 | 0.308 | 0.941 |
|  |  |  |  |  |  |  |  |  |
| 30 km | Water × mining | r | 0.711 | 0.933 | 0.632 | 0.351 | -0.475 | -0.281 |
|  |  | p | <0.001 | <0.001 | <0.001 | <0.001 | <0.001 | <0.001 |
|  | Water × agriculture | r | 0.353 | 0.863 | 0.529 | 0.291 | -0.284 | 0.124 |
|  |  | p | <0.001 | <0.001 | <0.001 | <0.001 | <0.001 | 0.127 |
|  |  |  |  |  |  |  |  |  |
| 4 0 km | Water × mining | r | 0.568 | 0.971 | 0.580 | 0.432 | -0.587 | -0.361 |
|  |  | p | <0.001 | <0.001 | <0.001 | <0.001 | <0.001 | <0.001 |
|  | Water × agriculture | r | 0.292 | 0.809 | 0.574 | 0.374 | -0.341 | -0.104 |
|  |  | p | 0.000 | <0.001 | <0.001 | <0.001 | <0.001 | 0.200 |
