# Supplementary material for: Buruli Ulcer Disease and Its Association with Land Cover in Southwestern Ghana
Source: PLoS Negl Trop Dis. 2015 Jun 19;9(6):e0003840. doi: 10.1371/journal.pntd.0003840 (PMC4474842; doi:10.1371/journal.pntd.0003840)
Supplement: S3 Table — (DOCX) [file pntd.0003840.s003.docx]

**Table S3.** The association of BU prevalence and the interaction between water and mining at different spatial extents using negative binomial regression models. The dependent variable is the natural logarithm -transformed number of BU cases in each village with the natural logarithm -transformed population in each village as an offset. The covariates are the percentages of land cover classes in a buffer in different distances.

| **Buffer radius** | **Covariates** | **β** | **95% CI** | | **p** | **AIC** |
| --- | --- | --- | --- | --- | --- | --- |
|  |  |  |  |  |  |  |
| 5 km | Urban | -0.164 | -0.246 | -0.081 | <0.001 | 593.09 |
|  | Water × mining | 0.245 | 0.032 | 0.459 | 0.025 |  |
|  | Grassland | 0.032 | -0.003 | 0.066 | 0.076 |  |
|  | Forest | 0.036 | 0.004 | 0.068 | 0.027 |  |
|  | Agriculture | 0.012 | -0.016 | 0.041 | 0.395 |  |
|  |  |  |  |  |  |  |
| 10 km | Urban | -0.506 | -0.674 | -0.338 | <0.001 | 573.23 |
|  | Water × mining | 0.809 | 0.048 | 1.571 | 0.037 |  |
|  | Grassland | 0.087 | 0.048 | 0.127 | <0.001 |  |
|  | Forest | 0.041 | 0.004 | 0.078 | 0.030 |  |
|  | Agriculture | 0.057 | 0.025 | 0.089 | 0.001 |  |
|  |  |  |  |  |  |  |
| 20 km | Urban | -1.115 | -1.436 | -0.794 | <0.001 | 551.52 |
|  | Water × mining | 2.940 | 1.208 | 4.672 | 0.001 |  |
|  | Grassland | 0.130 | 0.089 | 0.170 | <0.001 |  |
|  | Forest | 0.037 | -0.009 | 0.083 | 0.116 |  |
|  | Agriculture | 0.090 | 0.056 | 0.124 | <0.001 |  |
