# Supplementary material for: Buruli Ulcer Disease and Its Association with Land Cover in Southwestern Ghana
Source: PLoS Negl Trop Dis. 2015 Jun 19;9(6):e0003840. doi: 10.1371/journal.pntd.0003840 (PMC4474842; doi:10.1371/journal.pntd.0003840)

**S1 Fig.** **Seasonal-trend decomposition analysis of monthly BU cases**. The analysis was conducted with STL function in R package and BU data variable was decomposed into three components, i.e. trend, seasonal component and residual component. In x-axis, 2008.0 is January 2008, 2008.5 is July 2008, 2009.0 is January 2009, 2009.5 is July 2009, and 2010.0 is January of 2010.


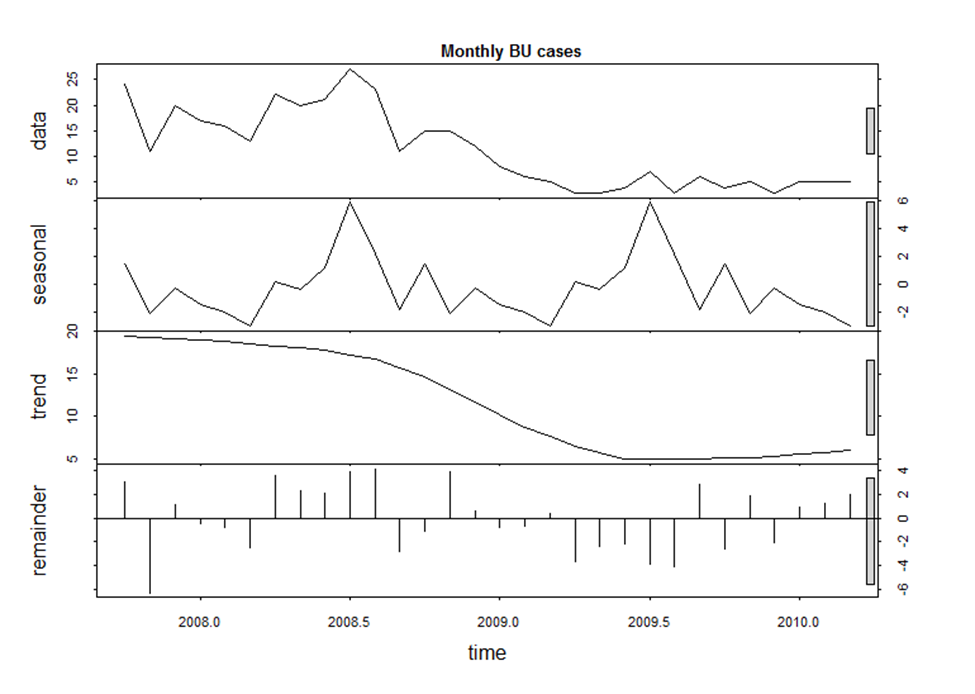

Supplement: S1 Fig — (DOCX) [file pntd.0003840.s004.docx]
