# Supplementary material for: Buruli Ulcer Disease and Its Association with Land Cover in Southwestern Ghana
Source: PLoS Negl Trop Dis. 2015 Jun 19;9(6):e0003840. doi: 10.1371/journal.pntd.0003840 (PMC4474842; doi:10.1371/journal.pntd.0003840)
Supplement: S2 Fig — (DOCX) [file pntd.0003840.s005.docx]

**S2 Fig.** The standardized Pearson residual and the standardized deviance residual from negative binomial regression model with a buffer radius of 30 km.
