# Supplementary material for: Buruli Ulcer Disease and Its Association with Land Cover in Southwestern Ghana
Source: PLoS Negl Trop Dis. 2015 Jun 19;9(6):e0003840. doi: 10.1371/journal.pntd.0003840 (PMC4474842; doi:10.1371/journal.pntd.0003840)
Supplement: S3 Fig — (DOCX) [file pntd.0003840.s006.docx]

**S3 Fig.** Comparison of land cover components before (plot A) and after (plot B) the assignment of unclassified pixels. (A) The number of pixels in each land cover class before reassigning unclassified pixels. (B) The number of pixels in each land cover class after reassigning unclassified pixels.
